# Breaking Object Correspondence Across Saccadic Eye Movements Deteriorates Object Recognition

**DOI:** 10.3389/fnsys.2015.00176

**Published:** 2015-12-21

**Authors:** Christian H. Poth, Arvid Herwig, Werner X. Schneider

**Affiliations:** ^1^Neuro-Cognitive Psychology, Department of Psychology, Bielefeld UniversityBielefeld, Germany; ^2^Cluster of Excellence Cognitive Interaction Technology, Bielefeld UniversityBielefeld, Germany

**Keywords:** saccade, visual stability, attention, object correspondence, transsaccadic memory

## Abstract

Visual perception is based on information processing during periods of eye fixations that are interrupted by fast saccadic eye movements. The ability to sample and relate information on task-relevant objects across fixations implies that correspondence between presaccadic and postsaccadic objects is established. Postsaccadic object information usually updates and overwrites information on the corresponding presaccadic object. The presaccadic object representation is then lost. In contrast, the presaccadic object is conserved when object correspondence is broken. This helps transsaccadic memory but it may impose attentional costs on object recognition. Therefore, we investigated how breaking object correspondence across the saccade affects postsaccadic object recognition. In Experiment 1, object correspondence was broken by a brief postsaccadic blank screen. Observers made a saccade to a peripheral object which was displaced during the saccade. This object reappeared either immediately after the saccade or after the blank screen. Within the postsaccadic object, a letter was briefly presented (terminated by a mask). Observers reported displacement direction and letter identity in different blocks. Breaking object correspondence by blanking improved displacement identification but deteriorated postsaccadic letter recognition. In Experiment 2, object correspondence was broken by changing the object’s contrast-polarity. There were no object displacements and observers only reported letter identity. Again, breaking object correspondence deteriorated postsaccadic letter recognition. These findings identify transsaccadic object correspondence as a key determinant of object recognition across the saccade. This is in line with the recent hypothesis that breaking object correspondence results in separate representations of presaccadic and postsaccadic objects which then compete for limited attentional processing resources (Schneider, 2013). Postsaccadic object recognition is then deteriorated because less resources are available for processing postsaccadic objects.

## Introduction

Accurate vision is spatially and temporally limited. Spatially, it is limited to the fovea, the center part of the eye’s retina which provides the highest visual resolution (e.g., Findlay and Gilchrist, 2003). The low resolution in the retinal periphery places a fundamental constraint on the visual exploration of the world: To view a potentially interesting object in the periphery with high acuity, one must bring it onto the fovea by making a fast saccadic eye movement. Temporally, online visual processing is limited to fixations, discrete episodes in which the eyes stand relatively still. Every saccade interrupts useful visual input and changes the retinal position and resolution of external objects. Nevertheless, humans perceive the visual world as stable across saccades (for reviews, see Bridgeman et al., 1994; Wurtz, 2008). Moreover, coping with most natural tasks demonstrates that humans sample and relate information on task-relevant objects across eye movements (Land and Tatler, 2009; Schneider, 2013). This implies that the visual system assesses *object correspondence* across fixations (Hollingworth et al., 2008; also called object continuity, Schneider, 2013), it assesses whether input from postsaccadic and presaccadic objects (apparently) comes from the same external object (Kahneman et al., 1992; Irwin and Andrews, 1996). Object correspondence is a prerequisite for updating presaccadic low-quality information on a peripheral object with postsaccadic foveal information on the same object (Henderson and Anes, 1994; Demeyer et al., 2009; Herwig and Schneider, 2014).

Transsaccadic object correspondence and updating are considered elementary for building a task-relevant representation of the visual environment, as they tie together the samples obtained from successive fixations (Schneider, 2013; Ganmor et al., 2015; Herwig, 2015a; Wolf and Schütz, 2015; Wurtz, 2015). However, it appears that signaling of object correspondence and updating can also strikingly impair perception. An object can be displaced during a saccade for up to a third of saccade amplitude without this being noticeable (Bridgeman et al., 1975). This form of transsaccadic change-blindness suggests that the postsaccadic object location updates and overwrites the presaccadic object location (Deubel et al., 1996). As a consequence, displacement perception suffers because only the postsaccadic object location remains available (Deubel et al., 1996).

How does the visual system assess object correspondence? Object correspondence is signaled if a test of the presaccadic object against the object after the saccade results in a match (Deubel et al., 1996; Tas et al., 2012). This notion is supported by a number of studies using the *blanking paradigm*, which breaks object correspondence by blanking a saccade target object during the saccade and delaying its reappearance until shortly after eye-landing (Deubel and Schneider, 1994; Deubel et al., 1996, 1998, 2002; the discussion in terms of object correspondence comes from Tas et al., 2012). Blanking improves accuracy in reporting transsaccadic displacements of the saccade target object considerably (Deubel and Schneider, 1994; Deubel et al., 1996). In addition, blanking improves accuracy in reporting transsaccadic changes of visual object features besides location (such as spatial frequency, Weiß et al., 2015; see, also Deubel et al., 2002). Together, these results indicate that blanking prevents updating and overwriting of the presaccadic object with the postsaccadic one. Both objects are compared and this allows to identify displacements (Deubel and Schneider, 1994; Deubel et al., 1996, 2002) and changes of other visual features (Weiß et al., 2015). Briefly occluding the postsaccadic object (Deubel et al., 2002) and changing its contrast-polarity (Tas et al., 2012) helps reporting displacements in a similar way as blanking. This suggests that breaking object correspondence in general prevents transsaccadic updating. Instead of one updated object representation, separate representations of the presaccadic and postsaccadic object should emerge (Deubel et al., 1996; Tas et al., 2012; Schneider, 2013).

Critically, the beneficial effects of breaking object correspondence for perceiving transsaccadic displacements and feature changes may come at costs in terms of postsaccadic object recognition. This hypothesis is based on the theory of “Task-dRiven visual Attention and working Memory” (TRAM, Schneider, 2013). TRAM follows the biased competition approach to attention (Desimone and Duncan, 1995) and the “Theory of Visual Attention” (Bundesen, 1990), assuming that visual objects compete for object recognition. Specifically, an object is recognized and becomes accessible (e.g., for report) if it enters capacity-limited visual working memory. An object can enter visual working memory if enough attentional processing resources (e.g., neurons, Bundesen et al., 2005) have been allocated to it. Object recognition is competitive because these processing resources are limited and have to be split among objects (Bundesen, 1990; Desimone and Duncan, 1995; Bundesen et al., 2005). Thus, the more objects take part in the competition, the less attentional processing resources are available for processing each individual object in service of object recognition. A central idea of TRAM is that the competition for object recognition is organized in discrete competition episodes of which eye fixations are a prominent case. Two kinds of objects participate in the competition. First, objects from the current episode, including those objects that have updated their corresponding counterparts from the preceding episode. Second, objects from the preceding episode for which no corresponding object was found in the current episode. Therefore, an object that has not been updated due to broken object correspondence introduces an additional competitor into the current competition episode. As a consequence, attentional processing resources must be split among more objects. This then cuts the resources for processing each individual object and thereby imposes costs on object recognition.

The present study aimed at testing the hypothesis that breaking object correspondence across the saccade deteriorates postsaccadic object recognition. Two experiments each used a different manipulation to break transsaccadic object correspondence and examined its effects on performance in a postsaccadic letter recognition task.

## Experiment 1

In Experiment 1, blanking was used to break transsaccadic object correspondence (Deubel et al., 1996; cf. Tas et al., 2012). Observers made a saccade to a peripheral object which was displaced during the saccade. The postsaccadic object appeared either immediately after the saccade (no-blank condition) or after a brief blank (blank condition). A single letter was presented simultaneously to and within the postsaccadic object and was terminated by a pattern mask. Both, displacement identification and postsaccadic object recognition performance were assessed. Observers reported displacement direction and letter identity in two different blocks of trials. If breaking object correspondence by blanking imposes costs on object recognition, then performance in reporting the postsaccadic letter should suffer in the blank condition compared to the no-blank condition. This predicted deterioration in object recognition is diametrical to the expected performance improvement for displacement identification (Deubel and Schneider, 1994; Deubel et al., 1996, 2002).

### Method

#### Observers

Sixteen observers (eight males, eight females) between 20 and 32 years (*Mdn* = 27 years) were paid to participate in Experiment 1. All had normal or corrected-to-normal vision (contact lenses) and gave written informed consent before the experiment. The type of experiment was approved by Bielefeld University’s ethics committee.

#### Apparatus and Stimuli

The experiment took place in a dimly lit room. Eye behavior was recorded by a video-based tower-mounted eye-tracker (Eyelink 1000, SR Research, Mississauga, ON, Canada) which was calibrated using a nine-point grid procedure and sampled observers’ right eyes at 1000 Hz. Observers’ heads were stabilized by forehead and chin rests, 71 cm from the 19”-CRT-screen (G90FB, ViewSonic, Brea, CA, USA) which ran with a resolution of 1024 × 768 pixels (at physical dimensions of 36 cm × 27 cm) and a refresh rate of 100 Hz.

The experiment was controlled by Experiment Builder (v1.10.1025). Stimulus luminance was measured using a MAVOLUX-digital luminance meter (Gossen, Nuremberg, Germany). Stimuli were black (<1 cd × m^-2^) special characters (§$&}/[μ∼) and letters (ABDGHJKLMNPRSTVX; 0.48° × 0.56°) in Arial font and a black plus-character (0.28° × 0.28°) was used as fixation cross. The saccade target object was a gray ellipse (29 cd × m^-2^; 0.7° × 1.26°). The white background had a luminance of 89 cd × m^-2^. Four different pattern masks were used, which consisted of rectangles (1.01° × 1.5°) filled with black scrambled lines of different widths.

#### Design and Procedure

**FIGURE 1:**
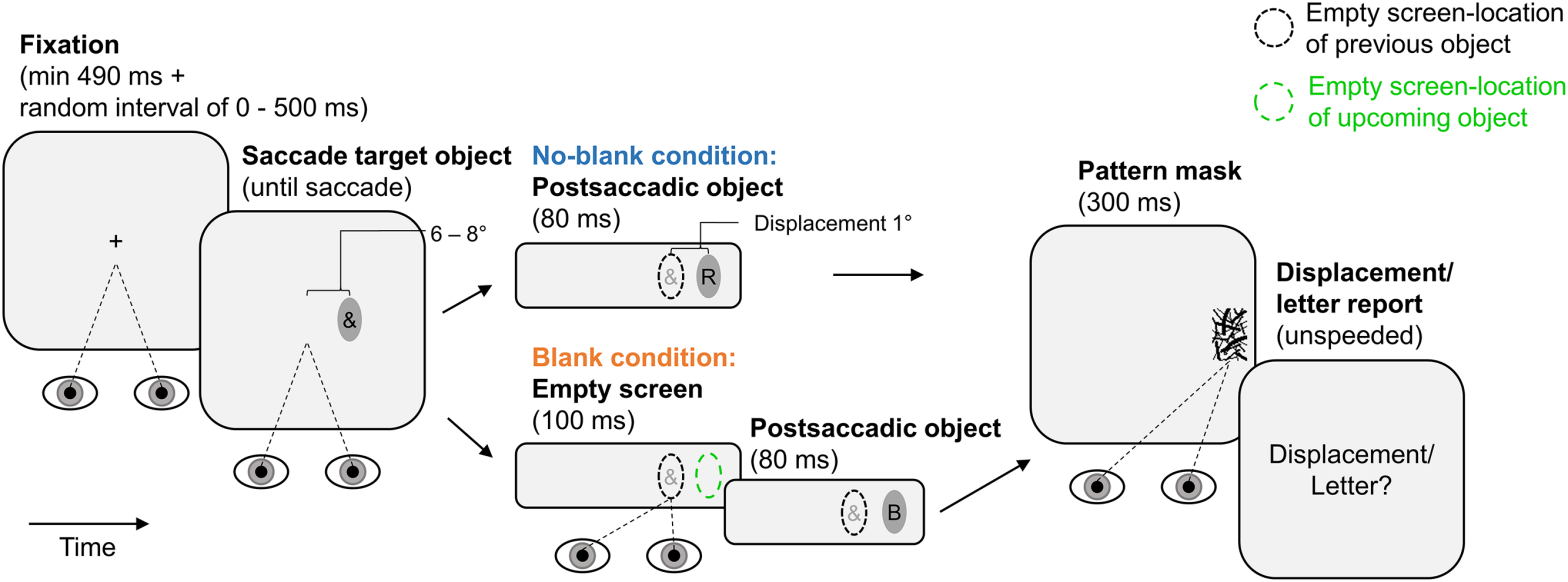
**Paradigm of Experiment 1.** Observers made a saccade to an elliptic object containing an irrelevant special character. The object was displaced during the saccade. The postsaccadic object contained a letter. It was shown for 80 ms (pattern-masked), either immediately after the saccade (no-blank condition) or after a 100 ms blank (blank condition). Displacement direction was reported in one report block, letter identity in the other. Ellipses of broken lines provide reference positions (they were not shown on the screen): A black ellipse of broken lines indicates the location of a previous object, a green ellipse of broken lines indicates the location of an upcoming object.

**Figure 1** illustrates the experimental paradigm. Each trial began with fixation of a central fixation cross (at least 490 ms continuous fixation plus a variable delay between 0 and 500 ms; trials were aborted and repeated if the fixation cross was not fixated). Afterward, the fixation cross was extinguished and an ellipse was shown as saccade target object, 6° or 8° from screen center in horizontal direction. This ellipse contained an irrelevant special character and was presented until the observer made a saccade to it (detected using velocity and acceleration thresholds of 30° × s^-1^ and 8000° × s^-2^). In the no-blank condition, the now empty ellipse was displaced for 1° during the saccade (with the next screen refresh after saccade detection). Initial position of the ellipse (6° or 8°, left or right to screen center) and displacement direction (left or right) were randomized across trials with equal numbers of occurrence in each condition. At the next screen refresh after eye-landing, a letter was shown within the ellipse for 80 ms and terminated by a pattern mask lasting for 300 ms. The letter was randomly drawn from the set of used letters (each letter occurred equally often in each blanking condition and report block; special characters were drawn analogously). The mask was drawn randomly from the set of used masks. After 500 ms, a response screen prompted observers to report letter identity or displacement direction using the keyboard (unspeeded forced choice; letter-keys or “F1” and “F12”, respectively). The next trial started after the report was made. The blank condition was identical to the no-blank condition except that an empty screen was shown during the saccade and lasted for another 100 ms from the screen refresh after the eye-landing. Trials of the two blanking conditions occurred in random order within report blocks. All observers performed two report blocks (order counterbalanced across sample) of 152 trials, the half of which belonging to the no-blank and the other to the blank condition. In these blocks, they either only reported displacement direction or only letter identity. Observers performed 16 training trials before each report block.

### Results

Trials were excluded from the analyses, if no saccade was made until 400 ms after onset of the saccade target object, saccade latency was below 100 ms (anticipatory saccades), or the saccade target object was missed by more than 2.5°. A total of 4.3% of the trials was discarded. Letter and displacement reports were each pooled across trials on which saccade target objects appeared 6° or 8° to the left or right of fixation (Deubel et al., 1996). They were also pooled across orders of displacement and letter report blocks because mixed analyses of variances (ANOVAs) showed that neither order nor the interaction of order and blanking conditions affected letter or displacement report performance, all *F*s < 3.167, all *p*s > 0.096.

**FIGURE 2:**
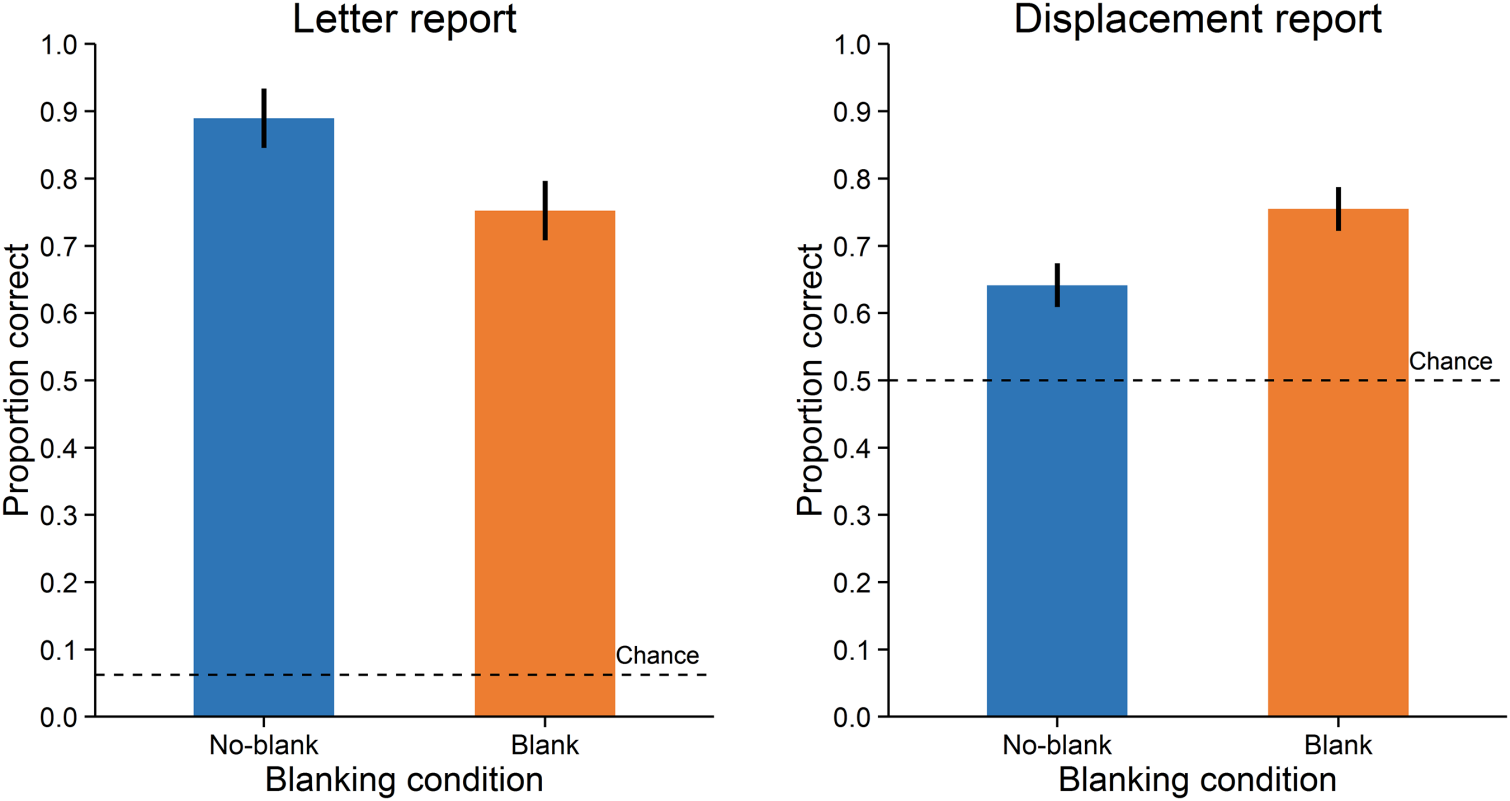
**Performance in Experiment 1.** Letter report performance **(left)** and displacement report performance **(right)**. Error-bars indicate 95% confidence intervals for within-subjects designs (Morey, 2008). Broken lines indicate chance level.

**FIGURE 3:**
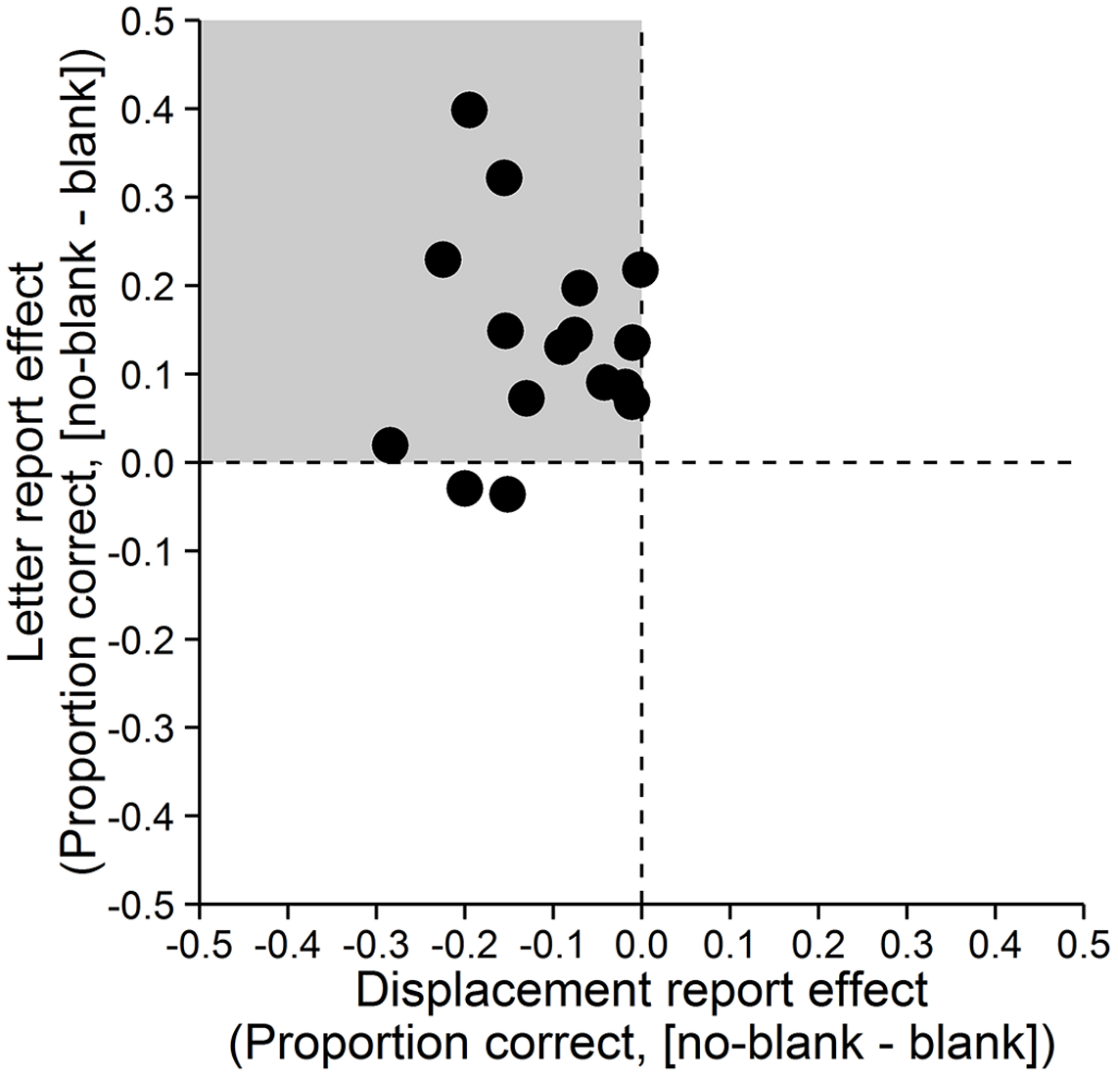
**Effects of blanking on letter and displacement reports for individual observers.** Differences between the no-blank and blank condition for both, displacement report (*x*-axis) and letter report (*y*-axis). Each point represents the value of one observer. The gray quadrant indicates the region in which points should fall if the effect of blanking on displacement report performance is in the opposite direction of the effect of blanking on letter report performance.

Accuracy was assessed as the proportion of correct responses. A paired-samples *t*-test with *d*_z_ (Cohen, 1988) as effect size showed that letter reports were significantly more accurate in the no-blank (*M* = 0.89, *SD* = 0.11) compared to the blank condition (*M* = 0.75, *SD* = 0.17), *t*(15) = 4.671, *p* < 0.001, *d*_z_ = 1.17, Bayes Factor (*BF*) = 108.271, (**Figure 2**, left; Bayes Factors were computed using the BayesFactor (0.9.10-2) package for R (3.0.3), cf. Rouder et al., 2009, values greater one support the alternative and values smaller one the null hypothesis). In contrast, displacement reports were significantly less accurate in the no-blank (*M* = 0.64, *SD* = 0.12) than in the blank condition (*M* = 0.75, *SD* = 0.16), *t*(15) = -5.238, *p* < 0.001, *d*_z_ = -1.31, *BF* = 284.724, (**Figure 2**, right). As evident from **Figure 3**, the effects of blanking on letter report performance and on displacement report performance were in opposite direction for most observers.

Not surprisingly, observers’ mean saccade latencies (i.e., the time between the onset of the saccade target object and saccade detection) did not differ significantly between the blanking conditions, both in the letter report block (no-blank: *M* = 132 ms, *SD* = 11 ms, blank: *M* = 133 ms, *SD* = 10 ms), *t*(15) = -1.756, *p* = 0.100, *d*_z_ = -0.44, *BF* = 0.893 and in the displacement report block (no-blank: *M* = 166 ms, *SD* = 21 ms, blank: *M* = 168, *SD* = 22 ms), *t*(15) = -0.858, *p* = 0.404, *d*_z_ = -0.21, *BF* = 0.352. The blanking conditions did not significantly differ in deviations of gaze positions from the postsaccadic object in the eye tracker’s first sample after the onset of the postsaccadic object (observers’ mean distance between gaze position and postsaccadic object), neither in the letter report block (no-blank: *M* = 1.14°, *SD* = 0.13°, blank: *M* = 1.18°, *SD* = 0.13°), *t*(15) = -1.730, *p* = 0.104, *d*_z_ = -0.43, *BF* = 0.864, nor in the displacement report block (no-blank: *M* = 1.16°, *SD* = 0.14°, blank: *M* = 1.18°, *SD* = 0.14°), *t*(15) = -0.545, *p* = 0.594, *d*_z_ = -0.14, *BF* = 0.291. Likewise, the blanking conditions did not significantly differ in variability of gaze positions in these samples of the eye tracker (observers’ standard deviation of distances between gaze position and postsaccadic object), neither in the letter report block (no-blank: *M* = 0.45°, *SD* = 0.09°, blank: *M* = 0.47°, *SD* = 0.08°), *t*(15) = -1.397, *p* = 0.183, *d*_z_ = -0.35, *BF* = 0.579, nor in the displacement report block (no-blank: *M* = 0.53°, *SD* = 0.11°, blank: *M* = 0.54°, *SD* = 0.10°), *t*(15) = -0.437, *p* = 0.669, *d*_z_ = -0.11, *BF* = 0.278.

### Discussion

Experiment 1 provides first support for the hypothesis that breaking object correspondence across the saccade impairs postsaccadic object recognition (Schneider, 2013). Recognition of a postsaccadic letter was deteriorated in the blank condition, where object correspondence was broken, compared to the no-blank condition, where it was not broken. In stark contrast, breaking object correspondence by blanking was beneficial for identifying transsaccadic object displacements. This beneficial effect of blanking on perception of transsaccadic object displacements replicates previous work and shows that the present blanking manipulation was effective (Deubel and Schneider, 1994; Deubel et al., 1996, 2002).

It is well-established that blanking breaks transsaccadic object correspondence (Tas et al., 2012) and prevents the updating and overwriting of presaccadic object information (Deubel and Schneider, 1994; Deubel et al., 1996, 2002; Weiß et al., 2015). However, some issues must be considered before we can conclude that the present deterioration in postsaccadic letter recognition was in fact due to broken object correspondence. First, the deterioration might have been due to the different temporal intervals between eye-landing and onset of the postsaccadic object in the two blanking conditions. Visual processing has been claimed to be enhanced immediately after saccades (Ibbotson and Krekelberg, 2011). Thus, processing of the postsaccadic letter might have been enhanced when the object was immediately visible after the saccade in the no-blank condition compared to when it appeared later in the blank condition. Second, the onset of the postsaccadic object was visible in the blank condition but was concealed by the saccade in the no-blank condition (e.g., Krock and Moore, 2015). Therefore, the deterioration might also stem from interference of this onset with recognition of the letter (as a form of masking; e.g., Enns and Di Lollo, 2000). Third, objects were always displaced during the saccade and this may have affected postsaccadic object recognition differently in the two blanking conditions. In line with these alternative explanations, one might suppose that object correspondence was broken in both blanking conditions, meaning it cannot account for the deteriorated postsaccadic letter recognition. This might have been the case because in both conditions a special character in the presaccadic object changed into a letter in the postsaccadic object (cf. Demeyer et al., 2010). To rule out these alternative explanations, Experiment 2 examined how postsaccadic letter recognition was affected by manipulating transsaccadic object correspondence in conditions with identical time courses and without any object displacements.

## Experiment 2

In Experiment 2, a change of contrast-polarity was used to break transsaccadic object correspondence (Tas et al., 2012). Observers made a saccade to a peripheral object which was black or white. The contrast-polarity of this object either stayed the same (no-change condition) or changed during the saccade (change condition) so that a black presaccadic object changed into a white postsaccadic one and vice versa. Similar to Experiment 1, a single letter appeared simultaneously to and within the postsaccadic object and was terminated by a pattern mask. In contrast to Experiment 1, however, both of these polarity-change conditions were identical in time course and there were no intrasaccadic object displacements. Observers’ only task was to report the postsaccadic letter. Now, if breaking object correspondence by changing contrast-polarity imposes costs on postsaccadic object recognition, then performance in reporting the postsaccadic letter should suffer in the change compared to the no-change condition.

### Method

#### Observers

Twelve observers (2 males, 10 females) were paid to take part in Experiment 2. They were between 21 and 31 years old (*Mdn* = 27), all had normal or corrected-to-normal vision (contact lenses) and gave written informed consent before the experiment. The type of experiment was approved by Bielefeld University’s ethics committee.

#### Apparatus and Stimuli

The apparatus and testing conditions in Experiments 1 and 2 were identical but not the same (i.e., the monitors were of the same model but were two different ones). Besides, a desktop-mounted video-based eye-tracker (Eyelink 1000, SR Research, Mississauga, ON, Canada) recorded eye behavior in Experiment 2.

Experiment 2 was controlled by the Psychophysics Toolbox (3.0.12; Brainard, 1997; Pelli, 1997; Kleiner et al., 2007) and Eyelink Toolbox (3.0.12; Cornelissen et al., 2002) extensions for MATLAB R2014b (The MathWorks, Natick, MA, USA). Stimuli were gray (67 cd × m^-2^) special characters (%#§&; 0.4° × 0.4°) and letters (ABDEFGHJKLMNOPRSTVXZ; 0.32° × 0.4°) in Arial font and saccade target objects were black (1 cd × m^-2^) or white (135 cd × m^-2^) ellipses (0.65° × 1.05°). The gray background had a luminance of 67 cd × m^-2^. A black square (0.1° × 0.1°) was used as central fixation stimulus. Ninety-nine pattern masks were algorithmically created for each observer and for both, black and white ellipses. This relatively large number of masks was chosen to minimize adaptation to the masks. The masks consisted of black or white rectangles (2° × 2°), each containing nine letters that were drawn randomly without replacement from the set of used letters. These letters were mirror-reversed and upside-down, they overlapped partially, and together covered an area of about 1° × 1° within a rectangle. For black rectangles the letters were white and for white rectangles they were black.

#### Design and Procedure

**FIGURE 4:**
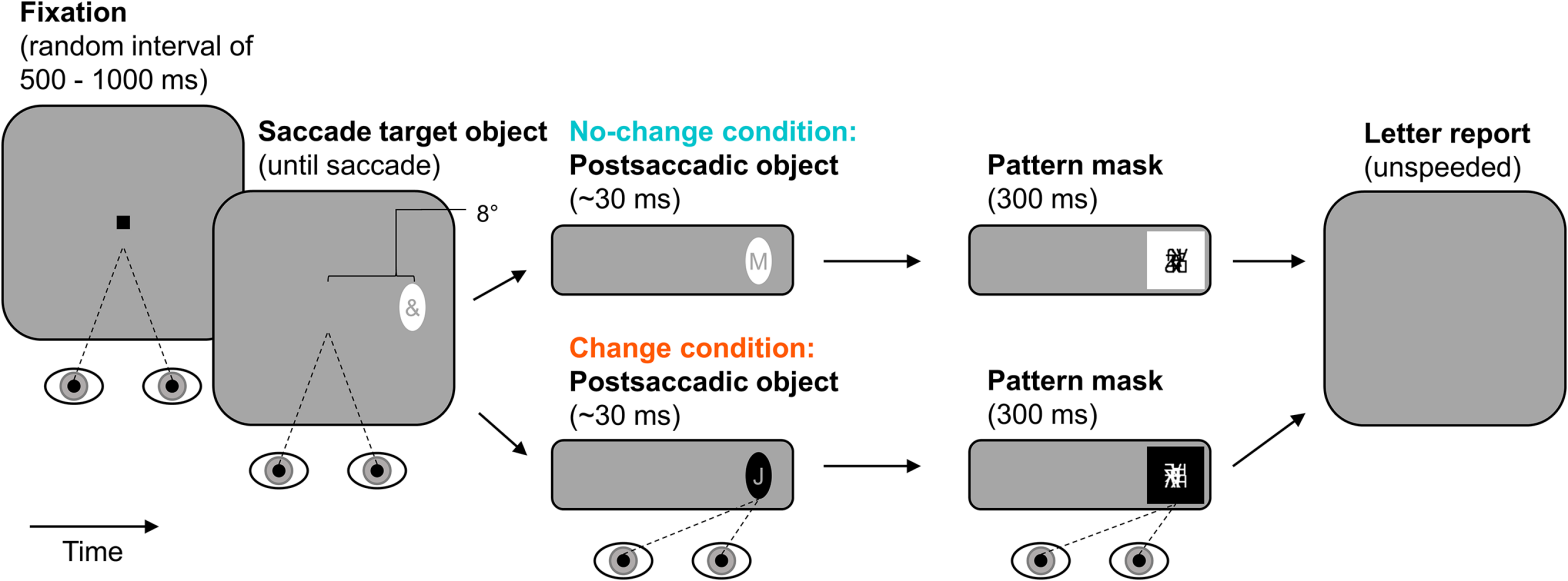
**Paradigm of Experiment 2.** Observers made a saccade to an elliptic object containing an irrelevant special character. The postsaccadic object was either of the same (no-change condition) or of the opposite contrast-polarity (change condition). It contained a letter and was visible for approximately 30 ms after the saccade (pattern-masked). Observers reported letter identity.

The experimental paradigm is illustrated in **Figure 4**. Observers started each trial by pressing the space-bar. In the beginning of a trial, observers fixated a central fixation stimulus for a random interval ranging from 500 to 1000 ms. Afterward, the fixation stimulus was extinguished and an ellipse was presented as saccade target object 8° to the left or right of screen center (randomized across trials with equal numbers of occurrence in each condition). The ellipse contained an irrelevant special character (randomly drawn from the set of used special characters) and stayed on screen until the observer made a saccade to it (detected using velocity and acceleration thresholds of 35° × s^-1^ and 9500° × s^-2^). This presaccadic ellipse was either black or white. The postsaccadic ellipse contained a letter (randomly drawn from the set of used letters) and appeared during the saccade, that is, on the next screen refresh after detection of saccade onset. In the no-change condition, the postsaccadic ellipse and the presaccadic ellipse were identical in their contrast-polarity. In the change condition, the postsaccadic ellipse was of the opposite contrast-polarity of the presaccadic ellipse. That is, a black presaccadic ellipse changed into a white postsaccadic one and vice versa. Whether presaccadic ellipses were black or white was randomized across trials but the number of occurrences was equal in the two polarity-change conditions. The postsaccadic ellipse was followed by a pattern mask of the same polarity. The mask was presented two or three screen refreshes after detection of saccade end so that the postsaccadic ellipse was visible after the saccade for 31 ms on average (*SD* = 3 ms). The mask was drawn randomly from the set of created masks and lasted for 300 ms. After that, the screen went blank and observers reported the letter using the keyboard (unspeeded forced-choice). They could start the next trial after 100 ms.

Observers performed 64 trials of each polarity-change condition in randomized order. Trials were aborted and repeated on a randomly chosen subsequent trial if observers failed to fixate the central fixation stimulus or if they missed the saccade target object by more than 2.5°. In this way, a total of 22.5% of the trials was repeated. Observers performed 32 training trials before the experiment.

### Results

Seven trials were excluded from analysis because saccade latency was below 100 ms or above 400 ms. Letter reports were pooled across trials on which saccade target objects appeared to the left or right of screen center (as for Experiment 1). They were also pooled across trials with different presaccadic ellipse polarities because a repeated-measures ANOVA indicated that neither presaccadic ellipse polarity nor its interaction with the two polarity-change conditions (i.e., no-change or change) affected letter report performance, both *F*s < 0.099, both *p*s > 0.758 (although distributions of proportions of correct responses were negatively skewed for both presaccadic ellipse polarities in the no-change condition).

**FIGURE 5:**
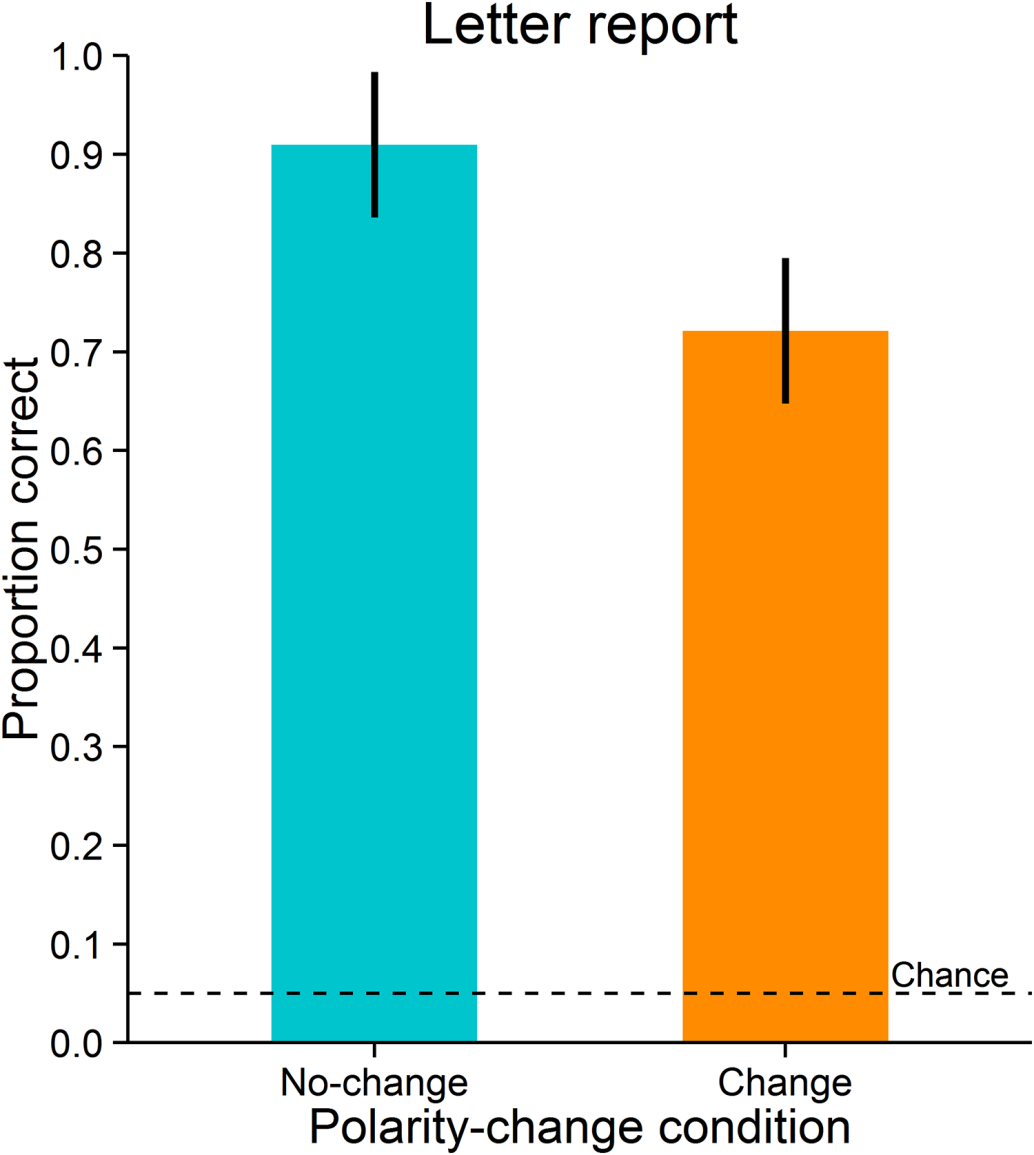
**Letter report performance in Experiment 2.** Error-bars indicate 95% confidence intervals for within-subjects designs (Morey, 2008). The broken line indicates chance level.

Accuracy was measured as the proportion of correct responses. Letter reports were significantly more accurate in the no-change condition (*M* = 0.91, *SD* = 0.15) than in the change condition (*M* = 0.72, *SD* = 0.20), *t*(11) = 3.989, *p* = 0.002, *d*_z_ = 1.15; *BF* = 21.223 (**Figure 5**). As can be expected, the two polarity-change conditions did not differ significantly in observers’ mean saccade latencies (no-change condition: *M* = 155 ms, *SD* = 20 ms; change condition: *M* = 155 ms, *SD* = 21 ms), *t*(11) = -0.494, *p* = 0.631, *d*_z_ = -0.14, *BF* = 0.319. Likewise, the conditions did not differ significantly in deviations of saccade landing positions from saccade target objects (observers’ mean distances between saccade landing positions and saccade target objects; no-change condition: *M* = 0.77°, *SD* = 0.19°; change condition: *M* = 0.79°, *SD* = 0.19°), *t*(11) = -1.665, *p* = 0.124, *d*_z_ = -0.48, *BF* = 0.846. Also, they did not differ significantly in variability of deviations of saccade landing positions from saccade target objects (observers’ standard deviations of distances between saccade landing positions and saccade target objects; no-change condition: *M* = 0.35°, *SD* = 0.07°; change condition: *M* = 0.35°, *SD* = 0.07°), *t*(11) = -0.216, *p* = 0.833, *d*_z_ = -0.06, *BF* = 0.293.

### Discussion

Experiment 2 provides further evidence that breaking transsaccadic object correspondence impairs postsaccadic object recognition (Schneider, 2013). Recognition of a postsaccadic letter was deteriorated in the change condition, where object correspondence was broken, compared with the no-change condition, where it was not broken. As such, the findings of Experiment 2 perfectly replicate and extend the findings from Experiment 1. Moreover, Experiment 2 also controlled for alternative interpretations of the findings of Experiment 1.

In Experiment 2, transsaccadic object correspondence was broken by changing contrast-polarity rather than by blanking. This allowed to keep the temporal interval between eye-landing and onset of the postsaccadic object constant in the two polarity-change conditions. Therefore, in contrast to Experiment 1, there were no differences in time course between conditions which could account for the differences in postsaccadic letter recognition. For this reason, two alternative explanations of the findings of Experiment 1 can be dismissed for the ones of Experiment 2. First, the differences in postsaccadic letter recognition did not result from enhanced processing immediately after saccades (Ibbotson and Krekelberg, 2011), because letter recognition would have been enhanced in both polarity-change conditions. Second, the differences did not result from interference of the onset of the postsaccadic object with letter recognition, because this onset happened during the saccade and likewise in both polarity-change conditions. Furthermore and again contrasting Experiment 1, there were no object displacements in Experiment 2. This excludes any differential effects of displacements between conditions. Both experiments had in common, however, that the presaccadic object contained an irrelevant special character which changed into a letter in the postsaccadic object. Although this change might have broken object correspondence (Demeyer et al., 2010), this cannot refer to the results of Experiment 2. The character change occurred in both polarity-change conditions and notwithstanding there was a pronounced effect of the polarity change on postsaccadic letter recognition. It has been shown previously that changing contrast-polarity is an effective tool to break transsaccadic object correspondence (Tas et al., 2012). Thus, even if the effect of changing contrast-polarity only added to the effect of changing the special character into the letter, it still demonstrates an effect of object correspondence on object recognition. Taken together, the findings of Experiment 2 therefore strongly argue that breaking object correspondence across the saccade deteriorates postsaccadic object recognition.

## General Discussion

We asked whether breaking object correspondence across the saccade impairs postsaccadic object recognition. The present findings indicate that this is the case. In both of our experiments, recognition of a postsaccadic letter was deteriorated when transsaccadic object correspondence was broken, compared with when it was not broken. Now we can ask which cognitive mechanisms might underlie these effects.

One possible interpretation of the present findings is that breaking transsaccadic object correspondence increases locational uncertainty of task-relevant information after the saccade. The precision of saccades is limited so that there is always variation in saccade landing positions. Therefore, to sample information on a saccade target object after a saccade, this object must be re-located (Hollingworth et al., 2008), even if it remained at its location across the saccade. Breaking transsaccadic object correspondence may hinder this re-location (and this might already happen during the saccade, Panouillères et al., 2013). Information on where to find task-relevant information after the saccade would then be less specific. This could impair postsaccadic object recognition, for instance because less attentional processing resources would be devoted to the location of the postsaccadic object.

Alternatively, intact transsaccadic object correspondence may provide computational savings which are lost in case object correspondence is broken. Specifically, new high-resolution foveal information on a postsaccadic object updates the representation of the corresponding presaccadic object (Tas et al., 2012; cf. Deubel and Schneider, 1994; Deubel et al., 1996, 2002). In contrast, if transsaccadic object correspondence is broken, then there is no presaccadic representation that can (or should) be updated with postsaccadic information. An entirely new representation must be created for the postsaccadic object. This additional requirement may delay processing of the postsaccadic object (such delays have for instance been found when monkeys had to adapt their smooth pursuit eye movements to postsaccadic motion patterns, Fallah and Reynolds, 2012). Such processing delays then deteriorate the postsaccadic recognition of objects and this is most prominent when postsaccadic objects are only briefly available (as in the current experiments).

These two interpretations suggest a close link between transsaccadic object correspondence and postsaccadic object recognition. However, they do not provide a mechanistic theory of the relationship between these processes. In contrast, TRAM (Schneider, 2013) may deliver a first step toward such a theory by proposing that *attentional weights* (Bundesen, 1990) are not only mediating competition for access to visual working memory across saccades but that they should also establish correspondence between presaccadic and postsaccadic objects.

Attentional weights represent the processing priority of objects by combining the task-driven and the intrinsic relevance of object features (Bundesen, 1990). Neuronally, attentional weights are assumed to exist in spatially organized priority maps in several brain areas (Bundesen et al., 2005; cf. Fecteau and Munoz, 2006; Cavanagh et al., 2010; Zelinsky and Bisley, 2015). Thus, attentional weights code for the feature-derived attentional priority of objects but also for their spatial location. With this combination of priority and location, attentional weights can provide a number of functions fundamental for human active vision. Within priority maps, attentional weights control saccade target selection (“where-to-look-next?”, Wischnewski et al., 2009, 2010; Schneider, 2013). This is a form of selection-for-action (Allport, 1987; Neumann, 1987). In addition, attentional weights govern the allocation of neuronal processing resources to objects in order to accomplish object recognition (Bundesen et al., 2005). This is selection-for-perception (covert visual attention). Selection-for-action and selection-for-perception are assumed to be tightly coupled (Schneider, 1995; Schneider and Deubel, 2002; cf. Irwin and Gordon, 1998) and attentional weights in priority maps may establish this coupling (Schneider, 2013; Herwig, 2015b). Furthermore, attentional weights (in this context called “attentional pointers”) can align presaccadic and postsaccadic information by keeping track of object locations across saccades (Cavanagh et al., 2010). This proposal is based on studies showing that the location sensitivity of neurons in some priority maps (i.e., the maps assumed to implement attentional weights, cf. Bundesen et al., 2005) is updated before saccades to accommodate impending saccade-induced changes of retinal locations (Duhamel et al., 1992). Along these lines, TRAM proposes that the attentional weight of a presaccadic and a postsaccadic object is used to test for object correspondence across saccades (Schneider, 2013). Object correspondence is then signaled if the attentional weight of the postsaccadic object matches the attentional weight that is predicted based on the presaccadic object. Thereby, the attentional weight could spatially route postsaccadic feature input to presaccadically created object representations in the process of transsaccadic updating. This may give rise to visual stability: the perception of a stable world despite the retinal image changes induced by saccades (e.g., Mathôt and Theeuwes, 2011).

In contrast, if object correspondence is broken, the visual system signals that a new object has appeared after the saccade (Kahneman et al., 1992; Irwin and Andrews, 1996). According to TRAM, the attentional weight of the presaccadic object is then encapsulated (i.e., retained with its current connection to presaccadic features) to protect the presaccadic object against being updated and overwritten by the new (non-corresponding) postsaccadic object. This encapsulated attentional weight competes with the attentional weights of postsaccadic objects. Neuronal processing resources are normalized over all present attentional weights (e.g., Bundesen et al., 2005; Poth et al., 2014). Instead of having all neuronal resources available for processing objects of the postsaccadic competition episode, some amount of resources is again (Schneider, 2013) or still (Petersen et al., 2012) allocated to the presaccadic object. In sum, TRAM proposes that breaking object correspondence across the saccade provokes attentional competition between presaccadic and postsaccadic objects. This attentional competition hypothesis provides one explanation why breaking object correspondence impaired postsaccadic object recognition in the present experiments. Testing the hypothesis may be an interesting avenue for future studies aiming to bridge research on transsaccadic object correspondence and on mechanisms of visual attention and object recognition.

## Conclusion

The present study shows for the first time that breaking object correspondence across the saccade deteriorates postsaccadic object recognition. This reveals a crucial role of object correspondence for vision across successive fixations and saccades. Natural human vision consists of a succession of fixations and saccadic eye movements. Therefore, classical theories of task-driven object recognition (and visual attention; Bundesen, 1990; Wolfe, 1994) should now take mechanisms of transsaccadic object correspondence into account.

## Author Contributions

CP, AH, and WS designed and planned the research. CP programmed the experiments and analyzed the data. CP, AH, and WS wrote the paper.

## Conflict of Interest Statement

The authors declare that the research was conducted in the absence of any commercial or financial relationships that could be construed as a potential conflict of interest.
